# Genomic profiling of *Streptococcus agalactiae* (Group B *Streptococcus*) isolates from pregnant women in northeastern Mexico: clonal complexes, virulence factors, and antibiotic resistance

**DOI:** 10.7717/peerj.19454

**Published:** 2025-05-22

**Authors:** Jose Manuel Vazquez-Guillen, Gerardo C. Palacios-Saucedo, Lydia Guadalupe Rivera-Morales, Amilcar Caballero-Trejo, Aldo Sebastian Flores-Flores, Juan Manuel Quiroga-Garza, Rocio Alejandra Chavez-Santoscoy, Jesus Hernandez-Perez, Silvia Alejandra Hinojosa-Alvarez, Julio Antonio Hernandez-Gonzalez, Maurilia Rojas-Contreras, Ricardo Vazquez-Juarez, Ramon Valladares-Trujillo, Cesar Alejandro Alonso-Tellez, Joaquin Dario Treviño-Baez, Miguel Angel Rivera-Alvarado, Reyes S. Tamez-Guerra, Cristina Rodriguez-Padilla

**Affiliations:** 1 Universidad Autonoma de Nuevo Leon, Facultad de Ciencias Biologicas, Laboratorio de Inmunologia y Virologia, San Nicolas de los Garza, Nuevo Leon, Mexico; 2 Division de Investigacion en Salud y Division de Auxiliares de Diagnostico, Unidad Medica de Alta Especialidad (UMAE) No. 25, Instituto Mexicano del Seguro Social, Monterrey, Nuevo Leon, Mexico; 3 Universidad Autonoma de Nuevo Leon, Facultad de Medicina, Departamento de Pediatria, Hospital Universitario “Dr. Jose Eleuterio Gonzalez”, Monterrey, Nuevo Leon, Mexico; 4 Departamento de Epidemiologia y Direccion de Educacion e Investigacion, Unidad Medica de Alta Especialidad No. 23 Hospital de Ginecologia y Obstetricia “Dr. Ignacio Morones Prieto”, Instituto Mexicano del Seguro Social, Monterrey, Nuevo León, Mexico; 5 Tecnologico de Monterrey, Escuela de Ingenieria y Ciencias, Instituto Tecnologico y de Estudios Superiores de Monterrey (ITESM), Monterrey, Mexico; 6 Laboratorio de Genomica y Bioinformatica, Centro de Investigaciones Biologicas del Noroeste S.C., La Paz, Baja California Sur, Mexico; 7 Laboratorio de Ciencia y Tecnologia de los Alimentos, Universidad Autonoma de Baja California Sur, La Paz, Baja California Sur, Mexico; 8 Coordinacion de Educacion e Investigacion en Salud, Hospital General de Zona No. 17, Instituto Mexicano del Seguro Social, Monterrey, Nuevo León, Mexico

**Keywords:** *Streptococcus agalactiae*, Pregnant people, Virulence factors, Drug resistance, Bacterial, Molecular epidemiology, Genomics

## Abstract

**Background:**

*Streptococcus agalactiae* (Group B *Streptococcus*, GBS) is an important pathogen associated with neonatal sepsis, pneumonia, and meningitis, which can be transmitted from colonized pregnant women to their newborns. This study aimed to determine the prevalence and characterize the genomic features of *S. agalactiae* isolates from pregnant women attending a referral hospital in Northeastern Mexico.

**Methods:**

Vaginal-rectal swabs were collected from pregnant women during routine prenatal care between April 2017 and March 2020. Whole-genome sequencing was conducted to determine sequence type (ST), clonal complex (CC), capsular polysaccharide (Cps) genotype, virulence factors, and antibiotic resistance genes through comparative genome analysis.

**Results:**

*S. agalactiae* colonization was detected in 51 (2.7%) of 1,924 pregnant women. The most common STs were ST8 (23.5%) and ST88 (15.7%). Cps genotyping showed high concordance between serological and molecular methods. Genes conferring resistance to tetracyclines (*tetM*, 60.1%) and macrolides (*mreA*, 100%) were identified. Key virulence factor genes, including *cylE*, *bca*, and *scpB*, were present in over 90% of the isolates.

**Conclusion:**

Although GBS colonization prevalence was low, genomic analysis revealed the genetic diversity of *S. agalactiae* in Northeastern Mexico, emphasizing the importance of molecular techniques for epidemiological surveillance and infection control.

## Introduction

*Streptococcus agalactiae* (*S. agalactiae*), commonly known as Group B Streptococcus (GBS), is an encapsulated Gram-positive bacterium that is part of the normal microbiota of the human gastrointestinal and genitourinary tracts (Furfaro, Chang & Payne, 2018). Despite its commensal nature, *S. agalactiae* is a significant pathogen, particularly in newborns, where it can cause invasive infections when colonization occurs in pregnant women during the later stages of pregnancy (Alsheim et al., 2024). In Latin America, *S. agalactiae* colonization rates range from 2% to 20.4%, with an estimated neonatal infection incidence of 0.3% to 1% (Palacios-Saucedo et al., 2017).

The pathogenicity of *S. agalactiae* is mediated by several virulence factors that enhance colonization and contribute to antimicrobial resistance (Burcham et al., 2019). Among these, the sialic acid capsular polysaccharide (Cps), encoded by the *cps* loci, is one of the most extensively studied. This polysaccharide is used to classify the bacterium into serotypes Ia, Ib, and II to IX and is known to facilitate immune evasion (Teatero et al., 2014). Other important virulence factors include laminin binding protein (Lmb), fibrinogen-binding proteins (Fbs), hypervirulent adhesin (HvgA), and alpha C protein (αC protein), all of which are associated with adherence and cell invasion (Bobadilla et al., 2021; Lacasse et al., 2022). The pili virulence factor, encoded by PI-1, PI-2a and PI-2b genes, confers resistance to antimicrobial peptides (Lu et al., 2015). Additionally, β-hemolysin/cytolysin, encoded by the *CylE* gene, acts as a pore-forming toxin (Shimizu et al., 2020). Several other virulence factors also contribute to immune evasion, cell adhesion and invasion, antimicrobial resistance, and toxin production, making them relevant to *S. agalactiae* pathogenesis (Rajagopal, 2009). Antibiotic resistance in *S. agalactiae* is an increasing concern. Macrolide resistance is conferred by the *mreA*, *mefA*, *mefE* and *ermB* genes, while tetracycline resistance is primarily associated with the *tetM* gene, which are commonly found in GBS (Mudzana, Mavenyengwa & Gudza-Mugabe, 2021; Liang et al., 2023).

Although numerous studies have examined the epidemiology and prevalence of *S. agalactiae* in pregnant women, further research is needed to characterize its molecular features to improve therapeutic and monitoring strategies (Delgado-Arévalo et al., 2020; Cabrera-Reyes et al., 2021; van Kassel et al., 2021). This study first aimed to determine the prevalence of *S. agalactiae* colonization in pregnant women attending a secondary referral hospital in Northeastern Mexico. Additionally, we sought to characterize the genetic diversity of *S. agalactiae* isolates by analyzing their sequence type (ST), clonal complex (CC), capsular polysaccharide (Cps) genotype, virulence factors, and antibiotic resistance genes.

## Materials and Methods

### Participants

Pregnant women attending prenatal care at the Hospital de Ginecologia y Obstetricia UMAE No. 23 of the Instituto Mexicano del Seguro Social (IMSS) were prospectively invited to participate between April 2017 and March 2020. Vaginal-rectal swab samples were collected from each participant following the American Society for Microbiology (ASM) guidelines for the detection and identification of GBS (Filkins et al., 2020). The swabbing procedure involved first sampling the lower vagina near the introitus, followed by the lower rectum through the anal sphincter using the same swab. The study was approved by the National Committee for Scientific Research of IMSS (approval number 2014-785-069), and all participants provided written informed consent.

## Wet lab procedures

### *S. agalactiae* identification and serotyping

*S. agalactiae* isolates were identified using standard microbiological and biochemical methods, including Gram staining, catalase testing, hippurate hydrolysis, the CAMP factor test with *Staphylococcus aureus* (ATCC25923), and culture in Strep B Carrot Broth (Hardy Diagnostics, Santa Maria, CA, USA). Lancefield group B confirmation was performed using the StrepPRO Streptococcal Grouping Kit (Hardy Diagnostics, Santa Maria, CA, USA). Capsular polysaccharide serotyping was conducted using the ImmuLex Strep-B Latex (Statens Serum Institute, Copenhagen, Denmark) latex agglutination test, which detects serotypes Ia, Ib, and II to IX.

### Whole-genome sequencing

*S. agalactiae* isolates were grown in 3 mL of Todd-Hewitt broth. Cellular pellets were harvested by centrifugation and treated with 180 μL of 50 mg/mL lysozyme for 60 min at 37 °C. Genomic DNA (gDNA) was extracted using the QIAamp DNA Mini Kit (Qiagen, Hilden, Germany) and quantified using the Quant-iT PicoGreen dsDNA Assay Kit (Thermo Fisher Scientific, Waltham, MA, USA) on a Qubit 2.0 Fluorometer (Thermo Fisher Scientific, Waltham, MA, USA). gDNA libraries were prepared using the Nextera DNA Flex Library Prep Kit (Illumina Inc., San Diego, CA, USA) and sequencing on a MiSeq (Illumina Inc., San Diego, CA, USA) instrument using the MiSeq Reagen Kit V2 (Micro Flow Cell 300 cycles; Illumina Inc., San Diego, CA, USA).

## Dry lab (*in silico*) procedures

### Bioinformatic data processing

Raw sequence reads were quality-assessed using FastQC (version 0.11.8) (Brown, Pirrung & Mccue, 2017). Draft genomes were assembled *de novo* with A5-miseq (Coil, Jospin & Darling, 2015). Assembly quality, including contig number and length, was evaluated with Quast (version 5.0.2) (Gurevich et al., 2013). Poor-quality assemblies were subjected to QC-Filtering pre-cleaning scripts. Genome annotation was performed using Prokka (version 1.12) (Seemann, 2014).

### Multi locus sequence typing (MLST)

MLST was performed using seven housekeeping genes (*adhP*, *pheS*, *atr*, *glnA*, *sdhA*, *glcK*, and *tkt*), with ST assigned *via* the MLST software (https://github.com/tseemann/mlst) (Jones et al., 2003) and CC determined using the PubMLST database (https://pubmlst.org/).

### Identification and mapping of capsular polysaccharide genotype, virulence factors, and resistance genes

Cps loci, virulence factor genes (*scpB*, *sodA*, *cspA*, *lmb*, *fbsA*, *fbsB*, *bca*, *hvgA*, *srr-1*, *srr-2*, *bib-A*, *dltA-D*, *ponA*, *cylE*, and *cfb*), and PI loci (PI-1, PI-2a, and PI-2b) were identified using BLAST homology with BRIG (version 0.95) (Alikhan et al., 2011). Antimicrobial resistance genes were detected using the Comprehensive Antibiotic Resistance Database (CARD) (Jia et al., 2017).

## Results

### *S. agalactiae* isolate characteristics and serotype distribution

A total of 1,924 pregnant women agreed to participate in the study, of whom 51 (2.7%) were colonized by *S. agalactiae*. Colonized women were 17 to 38 years old and in weeks 29 to 40 of gestation (Table 1). All isolates exhibited microbiological and biochemical characteristics consistent with *S. agalactiae*, including Gram-positive cocci morphology, catalase negativity, hippurate hydrolysis positivity, and CAMP factor test positivity. Additionally, all 51 isolates produced orange-pigmented colonies in Strep B Carrot Broth. Latex agglutination testing identified the following serotypes: Ia (15.7%), Ib (9.8%), II (19.6%), III (17.6%), IV (17.6%), V (9.8%), VIII (2.0%), and non-serotypeable (7.8%) (Table 2).

**Table 1:** Fifty-one *S. agalactiae* isolates and the basal characteristics of the pregnant women from which they were isolated.

*S. agalactiae* isolate	Age of pregnant women (years)	Gestation weeks	Urinary tract infections	Capsular polysaccharide serotype^a^
01	22	38	No	IV
02	21	40	No	V
03	31	32	No	III
04	24	29	Yes	III
05	37	39	No	V
06	27	37	No	Ia
07	25	34	ND	II
08	26	36	No	II
09	N/D	36	Yes	II
10	20	37	No	IV
11	26	36	Yes	III
12	30	35	No	IV
13	19	36	No	II
14	21	36	No	II
15	19	37	No	IV
16	28	35	No	Ib
17	28	37	No	II
18	32	38	No	II
19	32	34	Yes	IV
20	27	37	Yes	Ib
21	29	36	No	VIII
22	30	36	Yes	IV
23	23	35	Yes	Ia
24	38	37	Yes	V
25	30	35	Yes	IV
26	22	37	Yes	V
27	36	35	No	NST
28	28	37	Yes	IV
29	29	34	No	NST
30	37	34	Yes	Ib
31	31	35	Yes	II
32	17	37	Yes	NST
33	34	37	No	II
34	24	37	Yes	NST
35	32	37	Yes	Ia
36	33	37	No	Ib
37	22	34	Yes	III
38	26	36	Yes	III
39	30	37	No	Ib
40	28	34	Yes	III
41	22	34	Yes	III
42	31	37	No	II
43	19	37	No	Ia
44	21	36	No	IV
45	18	35	No	V
46	36	34	No	Ia
47	29	37	Yes	III
48	32	35	ND	III
49	20	37	No	Ia
50	22	35	No	Ia
51	30	37	No	Ia

**Notes:**

a Determined by commercial latex agglutination test ImmuLex Strep-B Latex (Statens Serum Institute, Copenhagen, Denmark).

NST, non-serotypeable; ND, not determined.

**Table 2:** Capsular polysaccharide serotype distribution of 51 *S. agalactiae* isolates.

	Total (*n* = 51)
Age (years)	28 [17–38]
Gestation weeks	36 [29–40]
*S. agalactiae* serotype^a^	
Ia	8 (15.7%)
Ib	5 (9.8%)
II	10 (19.6%)
III	9 (17.6%)
IV	9 (17.6%)
V	5 (9.8%)
VIII	1 (2.0%)
Non-serotypeable	4 (7.8%)

**Notes:**

Age and gestation weeks are presented as median and range.

Capsular serotype distribution is displayed in absolute frequencies and percentage.

a Determined by the commercial latex agglutination test ImmuLex Strep-B Latex (Statens Serum Institute, Copenhagen, Denmark).

### Assemblages generation and bioinformatics data analysis

**Figure 1:**
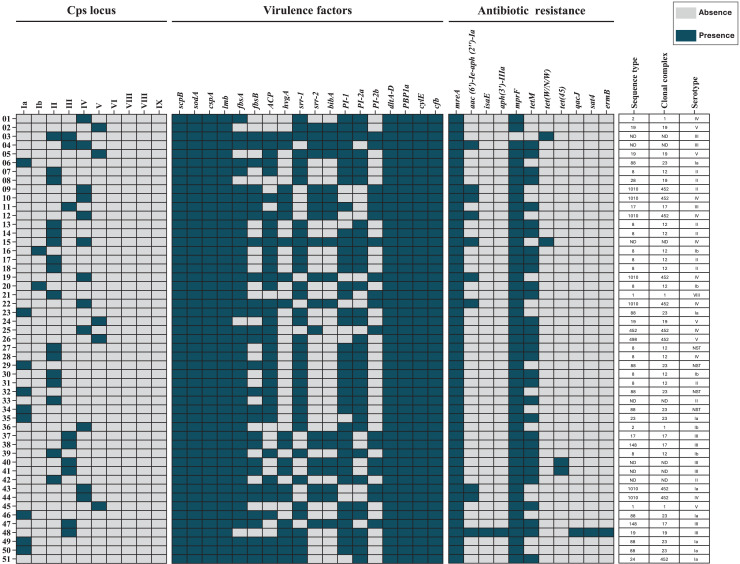
Heat map of the distribution of capsular polysaccharide (Cps) locus, virulence factors, and antibiotic resistance genes in 51 *S. agalactiae* genomes. Blue indicates gene presence, and gray indicates absence. Sequence types (STs), clonal complexes (CCs), and Cps serotypes for each isolate are shown. ND, not determined.

Draft genome assemblies were generated for all 51 isolates following quality control (Table S1). Genomic data are available under NCBI BioProjects PRJNA892112 and PRJNA551699. MLST analysis identified 13 distinct ST, grouped into six CC’s: CC12 (23.5%), CC452 (19.6%), CC23 (17.6%), CC19 (9.8%), CC1 (7.8%), and CC17 (7.8%). Seven isolates (13.7%) were unassigned to any CC (Fig. 1).

### Capsular polysaccharide genomic identification

Capsular polysaccharide loci were detected in 50 (98%) of the 51 isolates. Three isolates (5.9%) harbored loci for dual genotypes (II/III, III/IV, and II/IV). One isolate (2.0%) was non-genotypeable but had been serotyped as Ia. Genotyping and serotyping were concordant for 84.8% of isolates (Table 3).

**Table 3:** Concordance frequencies of capsular polysaccharide typing by latex agglutination *vs* Cps loci sequencing in 51 *S. agalactiae* isolates.

Cps serotypes identified by latex agglutination	Cps genotypes identified by sequencing										
	Ia	Ib	II	III	IV	V	VI	VII	VIII	IX	NGT
Ia	**6**				1						1
Ib		**2**	2		1						
II			**9**		1						
III				**9**							
IV			1		**8**						
V						**5**					
VI											
VII											
VIII			1								
IX											
NST	3		1								

**Note:**

NGT, non-genotypeable; NST, non-serotypeable.

### Virulence gene analysis

All 51 isolates (100%) carried the *scpB*, *sodA*, and *cspA* genes, which facilitate immune evasion. Genes associated with adherence and invasion were detected as follows: *lmb* in all isolates, *fbsA* in 46 (90.2%), and *fbsB* in 28 (54.9%). Other virulence genes included *bca* (37, 72.5%), *hvgA* (20, 39.2%), *srr-1* (36, 70.6%), *srr-2* (19, 37.3%), and *bibA* (18, 35.3%). Additionally, all isolates carried *dltA-D* and *ponA*, which mediate antimicrobial peptide resistance. Pili-encoding loci were distributed as follows: PI-1 in 39 (76.5%) isolates, PI-2a in 35 (68.6%), and PI-2b in 18 (35.3%). The cylE and cfb genes, encoding pore-forming toxins, were present in all 51 (100%) isolates (Table 4).

**Table 4:** Description and frequency of virulence factors identified by whole genome sequencing in 51 isolates of *S. agalactiae*.

Function^a^	Virulence factor	Description	Gene	Isolates^b^	Reference^c^
Immune evasion					
	ScpB	C5a peptidase	*scpB*	51 (100%)	U56908.1
	SodA	Superoxide dismutase	*sodA*	51 (100%)	KU598928.1
	CspA	Serine protease	*cspA*	51 (100%)	FJ752115.1
Host-cell adherence					
and invasion	Lmb	Laminin-binding protein	*lmb*	51 (100%)	AF062533.1
	FbsA	Fibrinogen-binding protein A	*fbsA*	46 (90.2%)	AJ437620.1
	FbsB	Fibrinogen-binding protein B	*fbsB*	28 (54.9%)	HQ267707.1
	αC protein	Alpha C protein	*bca*	37 (72.5%)	M97256.1
	HvgA	Hypervirulent GBS adhesin	*hvgA*	20 (39.2%)	CP020432.2
	Srr-1	Serine-rich repeat protein 1	*srr-1*	36 (70.6%)	CP010867.1
	Srr-2	Serine-rich repeat protein 2	*srr-2*	19 (37.3%)	AY669067.1
	BibA	Immunogenic bacterial adhesin	*bibA*	18 (35.3%)	FJ801035.1
Resistance to					
antimicrobial	PI-1	Pilus island 1	PI-1 locus	39 (76.5%)	EU929743.1
peptides	PI-2a	Pilus island 2a	PI-2a locus	35 (68.6%)	EU929327.1
	PI-2b	Pilus island 2b	PI-2b locus	18 (35.3%)	EU929402.1
	DltA-D	Alanylation of lipotechoic acid	*dltA-D*	51 (100%)	AJ291784.1
	PBP1a	Penicillin-binding protein 1a	*ponA*	51 (100%)	AY069949.2
Pore-forming					
toxins	β-H/C	β-hemolysin/cytolysin	*cylE*	51 (100%)	AF093787.2
	Cfb	CAMP factor	*cfb*	51 (100%)	EF694027.1

**Notes:**

a Classification from Rajagopal (2009).

b Values are shown in absolute frequencies (percentage)

c GeneBank ID sequence used as a reference for gene identification.

### Antimicrobial resistance gene identification

Antimicrobial resistance genes were categorized by identity level: “perfect” (exact match to CARD reference sequences) and “strict” (minor variations allowed) (McArthur et al., 2013). Perfect identity genes included *mreA*, found in all isolates (100%), and *aac(6′)-Ie-aph(2″)-Ia*, identified in 10 (19.6%) isolates. The *isaE* and *aph(3′)-IIIa* genes were each detected in one (2.0%) isolate. Strict identity matches included *mprF* in 50 (98.0%) isolates and *tetM* in 31 (60.1%). Less common genes, such as *tet(W/N/W)* and *tet(45)*, were each found in two (3.9%) isolates, while *qacJ*, *sat4*, and *ermB* were present in one (2.0%) isolate each (Table 5).

**Table 5:** Frequency of resistance genes identified in 51 isolates of *S. agalactiae*.

Identified genes^a^	Resistance to	Isolates^b^
**Perfect identity**		
*mreA*	Macrolides and clindamycin	51 (100%)
*aac (6′)-Ie-aph (2″)-Ia*	Aminoglycosides except streptomycin	10 (19.6%)
*isaE*	Lincosamides and pleuromutilins	1 (2.0%)
*aph(3′)-IIIa*	Aminoglycosides	1 (2.0%)
**Strict identity**		
*mprF*	Cationic antimicrobial peptides (CAMPs)	50 (98.0%)
*tetM*	Tetracycline	31 (60.1%)
*tet(W/N/W)*	Tetracycline (mosaic tetracycline resistance)	2 (3.9%)
*tet(45)*	Tetracycline	2 (3.9%)
*qacJ*	Quaternary ammonium compounds	1 (2.0%)
*sat4*	Streptothricins	1 (2.0%)
*ermB*	Macrolides	1 (2.0%)

**Notes:**

a Perfect identity refers to genes detected with an exact match to a CARD reference sequences. Strict identity allows some variation in similarity to the reference sequences (McArthur et al., 2013).

b Values are shown in absolute frequencies (percentage).

## Discussion

*S. agalactiae*, or GBS, is a leading cause of neonatal infections due to vertical transmission from mother to child during birth (Alsheim et al., 2024). In this study, we first determined the prevalence of *S. agalactiae* colonization among pregnant women attending a secondary referral hospital in Northeastern Mexico, identifying a colonization rate of 2.7% (51/1,924). Compared to other studies in Mexico, Cabrera-Reyes et al. (2021) reported a slightly higher prevalence of 4.3% (145/3,347) in pregnant women. Additionally, a meta-analysis by Reyna-Figueroa et al. (2007) documented a wide range of *S. agalactiae* prevalence rates (0.46% to 38%) across different regions of Mexico, with an average of 9.5%. Our findings fall within the lower range of this spectrum, suggesting potential regional differences in colonization rates or variations in detection methodologies. The observed discrepancies may be attributed to differences in population characteristics, clinical and environmental factors, sampling techniques, and microbiological detection methods. Furthermore, to gain deeper insight into the genetic diversity of *S. agalactiae* in this population, we characterized the isolates by analyzing their ST, CC, Cps genotype, virulence factors, and antibiotic resistance genes.

The most common serotypes in developed countries are Ia (31%), III (27%), V (19%), Ib (14%), and II (5%) (Hall et al., 2017). In our study, serotypes II (19.6%), III (17.6%), IV (17.6%), and Ia (15.7%) were the most frequent. The variation in serotype distribution may be influenced by geographical factors, as well as intra-country differences (Bobadilla et al., 2021). Additionally, we identified one pregnant woman colonized with serotype VIII, which is primarily reported in Asia (Lachenauer et al., 1999; Genovese et al., 2020). GBS capsular serotypes (Ia, Ib and II to IX) can be identified using latex agglutination and molecular methods such as PCR or sequencing. In this study, serotypes were identified in 47 (92.16%) of 51 isolates using serological tests, whereas capsular genotypes were identified in 50 (98.04%) of the 51 strains. A high agreement rate (82.98%, 39 of 47) was observed between the two methods, consistent with reports indicating >80% concordance between serological and molecular approaches (Yao et al., 2013; Brigtsen et al., 2015; Suhaimi et al., 2017). Some studies suggest the possibility of capsular polysaccharide switching, although clear evidence is lacking (Martins, Melo-Cristino & Ramirez, 2010; Bellais et al., 2012). These findings highlight the potential limitations of serological methods and underscore the importance of molecular approaches for accurate genotyping. The most frequent CCs associated with serotypes Ia, Ib, II, III, IV, and V are 23, 8, 22, 17, 459, and 1, respectively (McGee et al., 2021). Our results were consistent for serotypes Ia and III, but for serotype II, CC12 was predominant, while for serotypes IV and V, CC452 and CC19 were most common, respectively.

The GBS genome harbors various virulence factors. Consistent with previous studies, genes involved in immune evasion, such as *scpB*, *sodA* and *cspA*, were present in 100% of the isolates, with *sodA* commonly used as a housekeeping gene (Rosenau et al., 2007; Slotved et al., 2021; Koide et al., 2022). Genes associated with adhesion and invasion, such as *lmb*, were present in all isolates. Fibrinogen-binding protein genes were detected at rates of 54.9% for *fbsA*+/*fbsB*+, 35.3% for *fbsA*+/*fbsB*−, and 9.8% for *fbsA*−/*fbsB*−, aligning with previously reported data (Rosenau et al., 2007). The *bca* gene, which exhibits variable prevalence (21% to 88.6%), was detected in 72.5% of our isolates (Bobadilla et al., 2021; Lacasse et al., 2022). The *ssr-1* and *ssr-2* genes were found in 79.5% and 15.4% of isolates, respectively, compared to reported rates of 70.6% and 37.3%. The *bibA* gene, rarely detected in GBS, was present in 35.3% of our isolates, similar to the previously reported 34% (Lacasse et al., 2022). The *hvgA* gene, associated with hypervirulent strains, was identified in 39.2% of our isolates, exceeding the reported 12.8% (Burcham et al., 2019).

Pili are surface structures that contribute to *S. agalactiae* adhesion, host colonization, and biofilm formation, facilitating bacterial persistence in the host. In *S. agalactiae*, three pilus islands (PI-1, PI-2a, and PI-2b) have been identified, which can be present individually or in combination. Previous reports indicate the presence of PI-1 in 43.1%, PI-2a in 85.6%, and PI-2b in 14.4% of *S. agalactiae* isolates. In our study, the prevalence of PI-1 (76.5%) and PI-2b (35.3%) was higher, while PI-2a (68.8%) was lower compared to previous reports (Lu et al., 2015). While pili are primarily associated with bacterial adherence, previous studies suggest they may also play a role in antimicrobial peptide resistance by influencing bacterial interactions with the host immune system and antimicrobial compounds, although the precise mechanisms remain unclear (Rajagopal, 2009).

The *dltA-D* operon, which enhances bacterial resistance to host defenses, was present in 100% of our isolates, in agreement with studies indicating increased susceptibility in its absence (Armistead et al., 2019). The *cfb* gene, encoding pore-forming proteins, is universally present in GBS strains, with rare exceptions due to chromosomal deletions (Bobadilla et al., 2021; Creti et al., 2023). The *cylE* gene, which confers hemolytic properties and enhances virulence, was present in all hemolytic strains. Non-hemolytic strains, which are less virulent, constitute only 3% to 4% of reported isolates (Shimizu et al., 2020).

GBS resistance to clindamycin and erythromycin is primarily mediated by ribosomal methylation genes such as *ermB* or *ermA*. In this study, only one isolate (2.0%) carried *ermB*, while the *isaE* gene, associated with clindamycin resistance, was detected in isolates lacking *erm* genes. The *mreA* gene, encoding an antibiotic efflux pump that confers erythromycin resistance, was present in all isolates (Liu et al., 2023). Tetracycline resistance is commonly mediated by *tetM*, *tetL*, or *tetO*, with *tetM* being the most prevalent. In our study, 31 (60.1%) of isolates carried *tetM*, while *tet(W/N/W)* and *tet(45)* were detected in only 2 (2.0%) of cases (Gizachew et al., 2020).

Overall, our findings contribute important prevalence and genomic epidemiology data on *S. agalactiae* in northeastern Mexico. However, some limitations should be considered. First, the study was conducted at a single referral hospital, which may limit the generalizability of the findings to the broader population. Second, while whole-genome sequencing allowed for precise genetic characterization, phenotypic antimicrobial susceptibility testing was not performed to correlate resistance genes with actual resistance profiles. Lastly, the relatively low prevalence observed suggests that a larger sample size across multiple healthcare centers would be beneficial for a more comprehensive epidemiological assessment.

## Conclusion

This study determined the prevalence of *S. agalactiae* colonization among pregnant women attending a secondary referral hospital in northeastern Mexico, identifying a colonization rate of 2.7% (51/1,924), which falls within the lower range reported in previous studies across Mexico. To further understand the genetic diversity of *S. agalactiae* in this population, we characterized the isolates based on their ST, CC, Cps genotype, virulence factors, and antibiotic resistance genes. The predominant CC identified were CC452, CC23, and CC19. While Cps genotyping showed overall concordance between serological and molecular methods, some discrepancies were observed, including the detection of two Cps genotypes in the same isolate. All isolates harbored key virulence genes (*scpB*, *sodA*, *lmb*, and *fbsA*) at varying frequencies, and pili-encoding genes were present in distinct combinations. Additionally, all isolates carried *mreA* and *tetM*, highlighting the persistence of antimicrobial resistance determinants in *S. agalactiae*.

## Supplemental Information

10.7717/peerj.19454/supp-1 Supplemental Information 1 Quality characteristics of the assemblages generated from the gDNA libraries for whole genome sequencing of the 51 *S. agalactiae* isolates. The quality of the assemblies was evaluated with the Quast program (v 5.0.2; Gurevich A, et al. Bioinformatics 2013) and the annotation of the genome drafts was performed with Prokka (v1.12; Seemann T. Bioinformatics 2014).
^a^ CDS, coding sequences.

10.7717/peerj.19454/supp-2 Supplemental Information 2 Sequence Data BioProject PRJNA551699.

10.7717/peerj.19454/supp-3 Supplemental Information 3 Sequence Data BioProject PRJNA892112.

## Additional Information and Declarations

### Data Availability

The following information was supplied regarding data availability:

The meta-sequencing data are available at NCBI: PRJNA892112 and PRJNA551699.
