# Supplementary material for: Genomic profiling of Streptococcus agalactiae (Group B Streptococcus) isolates from pregnant women in northeastern Mexico: clonal complexes, virulence factors, and antibiotic resistance
Source: PeerJ. 2025 May 22;13:e19454. doi: 10.7717/peerj.19454 (PMC12103846; doi:10.7717/peerj.19454)
Supplement: Supplemental Information 1 — The quality of the assemblies was evaluated with the Quast program (v 5.0.2; Gurevich A, et al. Bioinformatics 2013) and the annotation of the genome drafts was performed with Prokka (v1.12; Seemann T. Bioinformatics 2014). a CDS, coding sequences. [file peerj-13-19454-s001.docx]

**Table S1.** Quality characteristics of the assemblages generated from the gDNA libraries for whole genome sequencing of the 51 *S. agalactiae* isolates.

| ***S. agalactiae* isolate** | **%GC** | **# contigs** | **Largest contig (bp)** | **Total length (bp)** | **N50 (bp)** | **L50** | **tARN** | **CDS^a^** |
| --- | --- | --- | --- | --- | --- | --- | --- | --- |
|  |  |  |  |  |  |  |  |  |
| 01 | 35.33 | 29 | 397.867 | 2,072,788 | 122.277 | 4 | 50 | 2.103 |
| 02 | 35.33 | 36 | 324.265 | 2,071,209 | 111.611 | 6 | 47 | 2.107 |
| 03 | 35.36 | 219 | 97.078 | 2,442,122 | 47.31 | 18 | 51 | 2.759 |
| 04 | 35.47 | 234 | 205.859 | 2,404,356 | 46.663 | 11 | 54 | 2.714 |
| 05 | 35.43 | 46 | 193.382 | 2,071,163 | 83.577 | 8 | 48 | 2.089 |
| 06 | 35.31 | 9 | 925.276 | 2,065,689 | 576.167 | 2 | 66 | 2.079 |
| 07 | 35.22 | 19 | 371.587 | 2,031,498 | 219.275 | 4 | 52 | 2.057 |
| 08 | 35.26 | 16 | 347.725 | 2,107,085 | 216.6 | 4 | 50 | 2.104 |
| 09 | 35.42 | 28 | 621.675 | 2,129,320 | 198.914 | 4 | 46 | 2.175 |
| 10 | 35.42 | 48 | 340.748 | 2,153,865 | 106.301 | 7 | 49 | 2.21 |
| 11 | 35.46 | 33 | 283.896 | 2,049,130 | 112.974 | 7 | 49 | 2.063 |
| 12 | 35.35 | 23 | 767.097 | 2,082,758 | 166.36 | 3 | 48 | 2.082 |
| 13 | 35.34 | 20 | 375.581 | 2,135,908 | 338.512 | 3 | 50 | 2.162 |
| 14 | 35.22 | 18 | 375.22 | 2,031,002 | 284.4 | 3 | 50 | 2.052 |
| 15 | 35.29 | 141 | 161.991 | 2,349,574 | 58.894 | 13 | 64 | 2.531 |
| 16 | 35.29 | 18 | 371.932 | 2,099,219 | 225.579 | 4 | 50 | 2.114 |
| 17 | 35.3 | 15 | 658.331 | 2,084,635 | 289.032 | 3 | 46 | 2.107 |
| 18 | 35.39 | 23 | 395.673 | 2,171,294 | 220.245 | 4 | 50 | 2.191 |
| 19 | 35.33 | 26 | 442.957 | 2,088,008 | 107.033 | 6 | 45 | 2.094 |
| 20 | 35.24 | 31 | 568.217 | 2,028,984 | 122.412 | 4 | 49 | 2.052 |
| 21 | 35.38 | 13 | 831.45 | 2,027,737 | 274.129 | 2 | 53 | 2.025 |
| 22 | 35.36 | 38 | 225.082 | 2,089,668 | 120.326 | 6 | 54 | 2.109 |
| 23 | 35.32 | 19 | 682.101 | 2,056,572 | 190.969 | 3 | 52 | 2.075 |
| 24 | 35.47 | 32 | 252.835 | 2,102,464 | 125.431 | 7 | 51 | 2.133 |
| 25 | 35.44 | 21 | 468.585 | 2,163,849 | 169.745 | 4 | 45 | 2.203 |
| 26 | 35.38 | 9 | 1,115,861 | 2,093,642 | 1,115,861 | 1 | 85 | 2.019 |
| 27 | 35.38 | 16 | 656.197 | 2,082,960 | 221.072 | 3 | 47 | 2.019 |
| 28 | 35.27 | 17 | 428.768 | 2,098,457 | 208.548 | 4 | 48 | 2.152 |
| 29 | 35.54 | 12 | 728.626 | 2,156,797 | 370.974 | 2 | 47 | 2.184 |
| 30 | 35.31 | 24 | 342.363 | 2,093,431 | 154.138 | 5 | 50 | 2.118 |
| 31 | 35.31 | 22 | 373.14 | 2,082,890 | 188.409 | 4 | 47 | 2.017 |
| 32 | 35.52 | 11 | 1,019,099 | 2,158,927 | 392.68 | 2 | 56 | 2.177 |
| 33 | 35.27 | 20 | 444.003 | 2,062,841 | 284.24 | 3 | 47 | 2.094 |
| 34 | 35.55 | 17 | 664.759 | 2,158,319 | 296.424 | 3 | 51 | 2.195 |
| 35 | 35.17 | 11 | 756.442 | 2,035,797 | 471.663 | 2 | 53 | 2.055 |
| 36 | 35.35 | 23 | 285.401 | 2,071,288 | 205.628 | 5 | 56 | 2.099 |
| 37 | 35.29 | 27 | 284.102 | 1,992,505 | 134.518 | 5 | 50 | 2.002 |
| 38 | 35.17 | 33 | 235.893 | 2,071,830 | 95.729 | 7 | 49 | 2.12 |
| 39 | 35.21 | 15 | 497.722 | 2,030,931 | 370.555 | 3 | 45 | 2.046 |
| 40 | 35.24 | 26 | 263.696 | 2,013,414 | 133.458 | 6 | 54 | 2.009 |
| 41 | 35.24 | 27 | 412.651 | 2,012,945 | 131.705 | 5 | 52 | 2.013 |
| 42 | 35.29 | 21 | 328.334 | 2,067,817 | 195.778 | 4 | 48 | 2.084 |
| 43 | 35.17 | 25 | 388.762 | 2,031,466 | 139.432 | 4 | 51 | 2.036 |
| 44 | 35.33 | 23 | 326.325 | 2,088,143 | 166.359 | 4 | 46 | 2.1 |
| 45 | 35.52 | 21 | 324.671 | 2,185,400 | 221.617 | 4 | 50 | 2.214 |
| 46 | 35.31 | 7 | 1,114,779 | 2,056,233 | 1,114,779 | 1 | 47 | 2.067 |
| 47 | 35.16 | 32 | 284.191 | 2,071,560 | 96 | 7 | 49 | 2.116 |
| 48 | 35.48 | 36 | 236.846 | 2,175,588 | 114.146 | 7 | 47 | 2.233 |
| 49 | 35.3 | 9 | 806.31 | 2,055,310 | 556.039 | 2 | 50 | 2.063 |
| 50 | 35.33 | 16 | 577.408 | 2,160,930 | 270.704 | 3 | 47 | 2.172 |
| 51 | 35.25 | 12 | 843.957 | 2,038,346 | 391.271 | 2 | 46 | 2.051 |

^a^CDS, Coding Sequences.

The quality of the assemblies was evaluated with the Quast program (v 5.0.2; Gurevich A, et al. Bioinformatics 2013) and the annotation of the genome drafts was performed with Prokka (v1.12; Seemann T. Bioinformatics 2014).
